# Comparison of circulating dendritic cell and monocyte subsets at different stages of atherosclerosis: insights from optical coherence tomography

**DOI:** 10.1186/s12872-017-0702-3

**Published:** 2017-10-18

**Authors:** Jianhui Zhuang, Yang Han, Dachun Xu, Guofu Zhu, Shekhar Singh, Luoman Chen, Mengyun Zhu, Wei Chen, Yawei Xu, Xiankai Li

**Affiliations:** 1 Department of Cardiology, Shanghai Tenth People’s Hospital, Tongji University School of Medicine, Shanghai, China; 2 Department of Pathology, Shanghai East Hospital, Tongji University School of Medicine, Shanghai, China; 3 Department of Cardiology, Shigatse People’s Hospital, Shigatse, Tibet China

**Keywords:** Dendritic cells, Monocytes, Plaque vulnerability, Optical coherence tomography

## Abstract

**Background:**

While specific patterns of circulating dendritic cells (DCs) and monocytes are associated with the incidence of coronary artery disease, the characterization of circulating DC and monocyte subsets in patients with different stages of atherosclerosis remains unclear.

**Methods:**

Forty-eight patients with unstable angina pectoris (UAP) diagnosed by angiography were enrolled. Likewise, 31 patients with ST-segment elevation myocardial infarction (STEMI) were enrolled and confirmed with the presence of thrombosis by angiography. Plaque features of 48 UAP patients were evaluated at the culprit lesions by OCT. Circulating myeloid DCs (mDCs), plasmacytoid DCs (pDCs) and monocyte subsets were analyzed using flow cytometry.

**Results:**

The proportions and absolute counts of mDC2s, which specifically express CD141 and possess the ability to activate CD8+ T lymphocytes, significantly decreased in patients with UAP and STEMI when compared with controls (0.08 × 10^4^ ± 0.05 × 104/ml and 0.08 × 10^4^ ± 0.06 × 104/ml vs. 0.11 × 10^4^ ± 0.06 × 104/ml, *p* = 0.027). On the other hand, patients with UAP and STEMI had significantly higher proportions and counts of Mon2 subsets. In the OCT subgroup, patients with thin-cap fibroatheroma (TCFA) had higher proportions and absolute number of Mon2 (11.96% ± 4.27% vs. 9.42% ± 4.05%, *p* = 0.034; 5.17 × 104/ml ± 1.92 × 104/ml vs. 3.53 × 104/ml ± 2.65 × 104/ml, *p* = 0.045) than those without TCFA. However, there was no remarkable difference in mDC2s between patients with and without TCFA.

**Conclusions:**

Circulating Mon2 appears to be a promising marker for the severity of atherosclerotic plaque.

**Electronic supplementary material:**

The online version of this article (10.1186/s12872-017-0702-3) contains supplementary material, which is available to authorized users.

## Background

During the development of coronary atherosclerosis, atherosclerotic plaque enlargement accompanied with narrowing of arterial luminal results in a series of chronic ischemic manifestations. More seriously, plaque instability and ensuing rupture with superimposed thrombi eventually lead to myocardial infarction and sudden death [1]. Thin-cap fibroatheroma (TCFA) is the most common phenotype of vulnerable plaque and is the precursor of plaque rupture. Although a greater understanding of lesion dimensions favors the clinical detection of TCFA in coronary artery disease (CAD) patients, coronary angiography alone could not provide comprehensive information on plaque morphology beyond luminal narrowing. Current clinical studies have suggested that optical coherence tomography (OCT) is one of the most preferred invasive approaches that allow high-resolution (10 μm) tomographic intra-arterial imaging [2, 3].

As professional antigen-presenting cells, monocytes and dendritic cells (DCs) possess the ability to recognize and present antigens to T cells [4]. Human circulating monocytes could be categorized into three subsets according to different expression of membrane receptors CD14 and CD16: a numerically dominant CD14++CD16- subset (Mon1), an intermediate CD14++CD16+ subset (Mon2), and a non-classical CD14 + CD16++ subset (Mon3) [5, 6]. Given the heterogeneity of circulating monocytes, the exact identity of atherosclerosis-related monocyte subsets deserves careful consideration. In this regard, some studies demonstrated that CD16 positive monocytes were associated with the incidence of myocardial infarction and in-stent restenosis [7, 8]. Furthermore, Rogacev et al. [9] observed that Mon2 subsets independently predicted the incidence of cardiovascular disease. Conversely, an earlier study by Shantsila et al. [10] did not find a marked correlation between monocyte subsets and the incidence of CAD. To our best knowledge, the relationship between monocyte subsets and atherosclerotic progress has not been examined.

Depending on the origin, location and function, DCs comprise two heterogeneous subpopulations. For human, plasmacytoid DCs (pDCs) express the surface marker blood dendritic cell antigen (BDCA)-2, while myeloid DCs (mDCs) could be categorized into mDC1 subsets with BDCA-1 and mDC2 subsets with BDCA-3 [11]. Recent reviews have helped solidify the knowledge of pDCs and mDCs in atherosclerosis [12]. Indeed, several clinical trials attempting to address whether circulating DC subsets correlate with the incidence and severity of CAD reached contradictory results [13, 14].

Toward this end, we sought to explore which subsets of DCs and monocytes are associated with the emergence, vulnerability and rupture of atherosclerotic plaques identified by coronary angiography and OCT.

## Methods

### Study population

Between April 2013 and July 2014, 112 subjects with acute chest pain as their first clinical manifestation undergoing coronary angiography and showing identifiable de novo culprit lesions in any native coronary artery were admitted at our hospital (Additional file 1: Figure S1). Among these individuals, 48 unstable angina pectoris (UAP) patients undergoing OCT were finally enrolled in the study. UAP was defined as angina at rest, accelerated angina or new-onset angina without elevation of cardiac markers [15]. Thirty-one ST-segment elevation myocardial infarction (STEMI) patients confirmed with the presence of acute thrombosis secondary to plaque rupture by angiography were also enrolled. The diagnosis of STEMI is clearly defined as ST-elevation in at least two contiguous leads >0.2 mV (2 mm) in chest leads and/or >0.1 mV (1 mm) in limb leads, and elevation of serum cardiac troponin-T (cTnT) level with at least one value above the 99th percentile upper reference limit [16]. Thirty-three subjects with possible cardiac etiology but free of luminal diameter narrowing ≥50% at coronary angiography served as controls.

The study was approved by Ethics Committee of Shanghai Tenth People’s Hospital and all individuals provided written informed concept before participation.

### Angiography and optical coherence tomography analysis

The located vessel, minimum lumen area and percent diameter stenosis of culprit lesion were measured. Culprit lesions were identified from a combination of electrocardiographic changes, echocardiographic findings and angiographic lesion morphologies.

Frequency domain Optical coherence tomography (OCT) imaging (C7XR, St. Jude, USA) was performed before any intervention and after intracoronary administration of nitroglycerin (0.2 mg) in UAP patients with a TIMI flow grade of 3. OCT images were digitalized and analysed offline according to the principals of OCT imaging described elsewhere [2, 17]. Two independent experienced clinicians who were blinded to the angiographic and clinical data analyzed the OCT images using validated criteria for plaque characterization **(**Additional file 2: Figure S2). Lipid cores at culprit lesions were defined as diffusely bordered, signal-poor regions. A fibrous cap was identified as a signal-rich homogenous region overlying a lipid core, which was characterized by a diffusely bordered, signal-poor region on the OCT image. Fibrous cap thickness was measured at the thinnest point in culprit plaques for three different times and the average value was calculated. The thinnest fibrous cap thickness was defined as the distance from the arterial lumen to the inner border of the lipid pool where the fibrous cap thickness is considered minimal in non-ruptured lipid-rich plaques. TCFA was defined as a plaque presenting lipid content for >90 degrees, and with thinnest part of the fibrous cap measuring <65 μm [18]. In the current research, there is no case enrolled with TCFA in all 3 vessels at the same time as inner quality control.

### Analysis of circulating DCs and monocytes by flow cytometry

Approximate 3–5 ml peripheral blood samples were taken at the day after admission from control group and UAP patients who had fasted overnight, while blood samples from STEMI patients were collected at the day of charge. It should be noted that all pheripheral blood samples from study population were obtained before coronary angiography. Peripheral leukocyte, polymorphonuclear granulocytes (PMNs), lymphocyte and monocyte counts were determined by Coulter Automated Hematology Analyzer (Beckman Coulter, USA). DC subsets from enrollments were analyzed using Blood Dendritic Cell Enumeration Kit (Miltenyi Biotec, Netherlands) [14]. Four-color flow cytometry (FACS Calibur, BD Biosciences, USA) was conducted using monoclonal antibodies against CD14, CD19, BDCA-1, BDCA-2 and BDCA-3 that were directly conjugated with fluorochromes. Dead cells, granulocytes, CD19+ B cells and CD14+ monocytes were excluded. The mDC1s were defined as cells positive for BDCA-1, while mDC2s were BDCA-3 immunopostive cells and pDCs were BDCA-2 immunopostive cells. To achieve the absolute mDC1, mDC2 and pDC count/ml, we set the above determined DC-proportions in relation to the total leucocyte count/ml. Monocyte subsets were analyzed in a whole-blood assay using 100 μl of whole blood as previously described [19]. FSC/SSC gate was positioned to exclude cell debris and granulocytes and monocytes were identified and gated in a CD45 (Miltenyi Biotec, Netherlands)/CD86 (BD Biosciences, USA) dot plots. Blood cells were simultaneously stained with anti-CD14-FITC (Miltenyi Biotec, Netherlands), anti-CD16-PE (Miltenyi Biotec, Netherlands) and anti-CCR2-APC (R&D Systems, UK) antibodies for 15 min at room temperature and analyzed by flow cytometry **(**Additional file 3: Figure S3). The flow cytometry for identifying DC and monocyte subsets was performed by one expert technician who was blind to the coronary angiography and OCT results.

### Biochemical analysis

Plasma levels of fasting glucose, total cholesterol and triglyceride, low-density lipoprotein cholesterol (LDL-C), high-density lipoprotein cholesterol (HDL-C) and high sensitivity C-reactive protein (hs-CRP), and serum levels of cTnT and creatine kinase-MB (CK-MB) were measured by colorimetric enzymatic assay systems (Roche MODULAR P-800, Swiss Confederation). Plasma levels of fibrinogen (Diagnostic Stago, France) and MMP9 (R&D System, USA) were measure using enzyme-linked immunosorbent assay by an automatic microplate reader (SpectraMaxi3, Molecular Devices, USA).

### Statistical anaylsis

Data were reported as mean ± standard deviation (SD) for continuous variables and as proportions for categorical variables. Student t test was undertaken to examine the differences between two groups. ANOVA test and Bonferroni correction were performed to compare the differences among controls, UAP patients and STEMI patients. The differences between categorical variables were determined using χ [2] test. Correlations between circulating DC and monocyte subsets and plaque characteristics were tested using the Pearson’s correlation test. A *p* value of <0.05 was considered statistically significant. Calculations were carried out using SPSS 14.0 software (SPSS Inc., USA).

## Results

### Baseline characteristics of patients

The baseline characteristics of enrollments are summarized in Additional file 4: Table S1. There was a male predominance in UAP and STEMI groups. The prevalence of smoking was higher in UAP and STEMI groups (35.4% and 51.6%, respectively) than in control group (12.1%). STEMI patients had higher levels of fasting glucose and triglyceride compared with healthy controls (fasting glucose: 7.4 ± 3.5 mmol/L vs. 5.5 ± 2.1 mmol/L; triglyceride: 3.0 ± 1.4 mmol/L vs. 1.7 ± 1.2 mmol/L). By definition, serum levels of cTnT and CK-MB were prominently higher in STEMI group as compared with control and UAP groups. Several established inflammatory markers such as hs-CRP, fibrinogen and MMP were markedly elevated in UAP and STEMI patients.

### Comparison of circulating DC and monocyte subsets among control, UAP and STEMI groups

Compared with controls and UAP patients, percent PMNs and PMNs/lymphocytes ratio were significantly higher, while a relative lower percentage of lymphocytes was observed in STEMI patients **(**Table 1 **)**. Although the percentages of mDC1s, mDC2s and pDCs were markedly lower in UAP and STEMI patients, the total numbers of mDC1s, mDCs and pDCs were comparable among three groups because total leukocyte count was greatly higher in UAP and STEMI patients. In line with percent mDC2s, the absolute number of mDC2s was lower in UAP and STEMI patients when compared with healthy controls (0.08 × 10^4^ ± 0.05 × 104/ml and 0.08 × 10^4^ ± 0.06 × 104/ml vs. 0.11 × 10^4^ ± 0.06 × 104/ml, *p* = 0.027). With regard to monocyte subsets, the percentage of total monocytes, as well as percent Mon1 subsets, was similar among three groups. The percentage of Monocyte 2 (Mon2) subsets was higher in UAP and STEMI patients than that in healthy controls (10.61% ± 4.17% and 13.38% ± 3.44% vs. 7.44% ± 2.40%, *p* < 0.001). In contrast, UAP and STEMI patients had lower percentages of Mon3 subsets as compared with healthy controls (6.97% ± 3.91% and 5.62% ± 4.96% vs. 10.29% ± 6.17%, *p* = 0.001). While the absolute number of Mon2 was markedly higher in UAP and STEMI patients (4.33 × 104/ml ± 2.39 × 104/ml vs. 7.22 × 104/ml ± 3.67 × 104/ml, p < 0.001), there was no significant difference in the absolute number of Mon3 among three groups (control, UAP and STEMI group: 4.43 × 104/ml ± 3.34 × 104/ml, 3.14 × 104/ml ± 2.20 × 104/ml and 3.32 × 104/ml ± 2.02 × 104/ml, *p* = 0.187) due to the gradually elevated number of circulating total monocytes.

**Table 1:** Total counts and proportions of peripheral blood cells in three groups

	Control (*n* = 33)	UAP (*n* = 48)	STEMI (*n* = 31)	p value
WBC, ×10^6^/ml	6.47 ± 1.72	6.99 ± 1.97	8.88 ± 2.41	*<0.001*
PMN, % WBC	58.10 ± 8.81	60.74 ± 9.64	76.51 ± 10.59	*<0.001*
Lym, % WBC	32.48 ± 7.84	32.11 ± 9.43	16.65 ± 9.44	*<0.001*
PMN/Lym ratio	1.99 ± 0.96	2.26 ± 1.45	7.02 ± 5.66	*<0.001*
Mon, % WBC	6.60 ± 1.17	5.83 ± 1.33	6.18 ± 2.50	0.179
mDC1s, % WBC	0.29 ± 0.05	0.22 ± 0.05	0.19 ± 0.04	*<0.001*
mDC2s, % WBC	1.69 × 10^−2^ ± 0.73 × 10^−2^	1.11 × 10^−2^ ± 0.42 × 10^−2^	0.81 × 10^−2^ ± 0.52 × 10^−2^	*<0.001*
mDCs, % WBC	0.31 ± 0.05	0.23 ± 0.05	0.20 ± 0.04	*<0.001*
pDCs, % WBC	0.20 ± 0.05	0.16 ± 0.03	0.14 ± 0.02	*<0.001*
mDC1s, ×10^4^/ml	1.89 ± 0.66	1.60 ± 0.70	1.73 ± 0.58	0.151
mDC2s, ×10^4^/ml	0.11 ± 0.06	0.08 ± 0.05	0.08 ± 0.06	*0.027*
mDCs, ×10^4^/ml	2.00 ± 0.69	1.68 ± 0.73	1.81 ± 0.62	0.126
pDCs, ×10^4^/ml	1.19 ± 0.43	1.15 ± 0.37	1.20 ± 0.35	0.862
Mon1, % monocytes	82.37 ± 6.31	82.36 ± 5.54	81.03 ± 5.91	0.564
Mon2, % monocytes	7.44 ± 2.40	10.61 ± 4.17	13.38 ± 3.44	*<0.001*
Mon3, % monocytes	10.29 ± 6.17	6.97 ± 3.91	5.62 ± 4.96	*0.001*
Mon1, ×10^5^/ml	3.45 ± 0.95	3.42 ± 1.13	4.41 ± 1.97	*0.007*
Mon2, ×10^4^/ml	3.06 ± 1.13	4.33 ± 2.39	7.22 ± 3.67	*<0.001*
Mon3, ×10^4^/ml	4.43 ± 3.34	3.14 ± 2.20	3.32 ± 2.02	0.187

Values are mean ± SD

Abbreviations: *DC* dendritic cell, *Lym* lymphocyte, *mDC* myeloid dendritic cell, *Mon* monocyte, *pDC* plasmacytoid dendritic cell, *PMNs* polymorphonuclear granulocytes, *WBC* white blood cell. *P*-values of risk factors with significance are presented as italic form

Because of the significant differences in the history of smoking and the plasma levels of fasting glucose and triglyceride among three groups, we investigated whether circulating DC and monocyte subsets were associated with these established risk factors. There were no discrepancies in the percentage and count of DC and monocyte subsets among subjects with and without histories of smoking **(**Additional file 5: Table S2). Likewise, the percentage and count of DC and monocyte subsets were not correlated with the plasma levels of fasting glucose and triglyceride **(**Additional file 6: Table S3).

Considering the roles of DCs and monocytes in inflammation and myocardial infarction, we examined the correlation of circulating DC and monocytes subsets with inflammatory and myocardial necrotic markers. Hs-CRP was positively associated with the percentage and absolute number of Mon1 **(**Additional file 7: Table S4). Similarly, plasma levels of MMP9 were significantly associated with the percentage and absolute number of Mon1 and Mon2. Furthermore, Mon1, Mon2 and mDCs, especially mDC2s, were closely related to serum levels of cTnT and CK-MB, both of which reflected the severity of myocardial infarction.

### Angiography and OCT findings

Of UAP patients, 20 (41.7%) were diagnosed as TCFA confirmed by OCT. The angiography and OCT data are shown in Table 2. Patients with TCFA had thinner fibrous cap (48.5 μm ± 10.9 μm vs. 119.6 μm ± 22.2 μm, *p* < 0.001) and larger lipid core (arc of lipid core, 252.0° ± 77.8° vs. 170.4° ± 67.7°, p < 0.001) compared with patients without TCFA. Plaque calcification was detected more frequently in patients with TCFA (45.0% vs. 17.9%, *p* = 0.041). No significant difference was found when comparing patients with and without TCFA in reference to the rate of multivessel disease, culprit lesion distribution, minimal lumen area and percentage of diameter stenosis.

**Table 2:** Angiographic and OCT finding

	Patients with TCFA (*n* = 20)	Patients without TCFA (*n* = 28)	p value
Age, yrs	62.4 ± 7.7	60.8 ± 9.4	0.541
Male	14 (70.0)	25 (89.3)	0.091
Multivessel disease	6 (30.0)	7 (25.0)	0.701
Culprit vessel			
LAD	10 (50.0)	10 (35.7)	0.457
LCX	6 (30.0)	8 (28.6)	
RCA	4 (20.0)	10 (35.7)	
Fibrous cap thickness, μm	48.5 ± 10.9	119.6 ± 22.2	*<0.001*
Arc of lipid core, degrees	252.0 ± 77.8	170.4 ± 67.7	*<0.001*
Calcium	9 (45.0)	5 (17.9)	*0.041*
MLA, mm^2^	4.5 ± 2.5	5.5 ± 2.7	0.215
Relative stenosis, %	56.8 ± 13.4	48.4 ± 16.2	*0.064*

Values are mean ± SD or n (%)

Abbreviations: *LAD* left anterior descending coronary artery, *LCX* left circumflex coronary artery, *MLA* minimum lumen area, *OCT* optical coherence tomography, *RCA* right coronary artery, *TCFA* thin-cap fibroatheroma. *P*-values of risk factors with significance are presented as italic form

### Relation of circulating DCs and monocytes with plaque characteristics

In a subgroup analysis of 48 UAP patients with plaque morphologies determined by OCT, we found that patients with TCFA had higher proportions of Mon2 subsets (11.96% ± 4.27% vs. 9.43% ± 5.15%, *p* = 0.035) and lower proportions of Mon3 subsets (4.93% ± 3.22% vs. 8.43% ± 3.75%, *p* = 0.001) as compared with patients without TCFA **(**Table 3 **)**. In UAP patients with TCFA, the absolute number of Mon2 was significantly higher than those without TCFA (5.17 × 104/ml ± 1.92 × 104/ml vs. 3.53 × 104/ml ± 2.65 × 104/ml, *p* = 0.045), whereas the counts of Mon3 did not differ between two groups (2.23 × 104/ml ± 1.44 × 104/ml vs. 3.66 × 104/ml ± 2.16 × 104/ml, *p* = 0.059). There were no significant differences in the percent PMNs and lymphocytes, PMNs/lymphocytes ratio, the absolute and percent DC subsets, and the proportion of Mon1 subsets between patients with and without TCFA. The correlation between plaque characteristics determined by OCT and circulating DCs and monocytes is summarized in Additional file 8: Table S5. The percentage of Mon3 was positively correlated with fibrous cap thickness (*r* = 0.409, *p* = 0.004) and inversely correlated with arc of lipid core (*r* = −0.353, *p* = 0.014). The absolute number of Mon2 was inversely correlated with fibrous cap thickness (*r* = −0.383, p = 0.014) and positively correlated with arc of lipid core (*r* = 0.413, *p* = 0.001). However, the percentages and numbers of circulating DCs and Mon1 did not differ in UAP patients with and without calcified plaques **(**Additional file 9: Table S6).

**Table 3:** Total counts and proportions of peripheral blood cells in patients with and without TCFA

	Patients with TCFA (n = 20)	Patients without TCFA (n = 28)	p value
WBC, ×10^6^/ml	6.88 ± 1.98	7.12 ± 1.98	0.690
PMN, % WBC	59.83 ± 9.45	61.33 ± 9.88	0.613
Lym, % WBC	32.75 ± 8.94	31.70 ± 9.87	0.717
PMN/Lym ratio	2.22 ± 1.69	2.29 ± 1.31	0.883
Mon, % WBC	5.83 ± 1.25	5.82 ± 1.41	0.989
mDC1s, % WBC	0.24 ± 0.05	0.21 ± 0.04	0.134
mDC2s, % WBC	1.08 × 10^−2^ ± 0.39 × 10^−2^	1.13 × 10^−2^ ± 0.44 × 10^−2^	0.664
mDCs, % WBC	0.25 ± 0.05	0.23 ± 0.05	0.151
pDCs, % WBC	0.15 ± 0.03	0.16 ± 0.03	0.285
mDC1s, ×10^4^/ml	1.63 ± 0.61	1.57 ± 0.76	0.791
mDC2s, ×10^4^/ml	0.08 ± 0.04	0.08 ± 0.05	0.605
mDCs, ×10^4^/ml	1.71 ± 0.64	1.66 ± 0.79	0.825
pDCs, ×10^4^/ml	1.07 ± 0.30	1.22 ± 0.41	0.173
Mon1, % monocytes	82.84 ± 6.17	82.02 ± 5.15	0.754
Mon2, % monocytes	11.96 ± 4.27	9.43 ± 5.15	*0.035*
Mon3, % monocytes	4.93 ± 3.22	8.43 ± 3.75	*0.001*
Mon1, ×10^5^/ml	3.25 ± 0.96	3.52 ± 1.22	0.470
Mon2, ×10^4^/ml	5.17 ± 1.92	3.53 ± 2.65	*0.045*
Mon3, ×10^4^/ml	2.23 ± 1.44	3.66 ± 2.16	0.059

Values are mean ± SD

Abbreviations as Table 1 and Table 2. *P*-values of risk factors with significance are presented as italic form

**Fig. 1:**
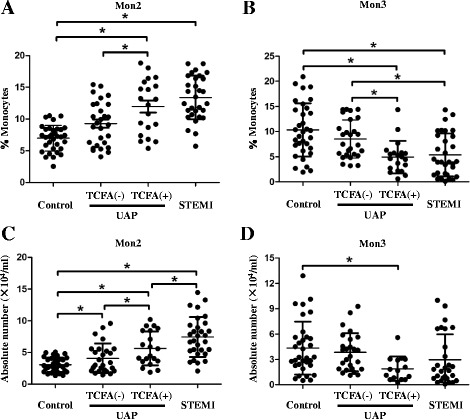
Comparison of CD14++CD16+ Mon2 and CD14 + CD16++ Mon3 subsets at different stages of atherosclerosis. Comparison of the percentages of Mon2 (**a**) and Mon3 (**b**) subsets among four groups. Comparison of the absolute numbers of Mon2 (**c**) and Mon3 (**d**) among four groups. * *P* < 0.05

Furthermore, according to the initiation and progression of atherosclerosis, the enrollments were divided into four groups as follows: control, non-TCFA, TCFA and STEMI groups. In this context, Fig. 1 shows a gradual increase in the proportion and absolute number of Mon2 subsets and a gradual decrease in the proportion of Mon3 subsets as the atherosclerosis worsen.

## Discussion

In the present study, we investigated the association of circulating inflammatory cells with morphological characteristics of atherosclerotic plaque observed by coronary angiography and OCT. The results herein demonstrated that elevated CD14++CD16+ Mon2 subsets were associated with plaque vulnerability and rupture determined by angiography and OCT.

There were a growing number of researches regarding the relationships among circulating DCs, inflammation and their effects on coronary plaque, whereas conflicting results were reported. While Yilmaz et al. [13] and Kretzschmar et al. [20] observed markedly decreases in circulating mDCs, pDCs and total DCs in stable CAD and STEMI patients, Wen et al. [21] found a reduction in circulating mDCs, but not pDCs, in patients with unstable CAD. The disparate findings among published literatures may be explained as follow. Total DCs make up only approximate a 0.4 percentage of leukocytes in peripheral blood. It is thus conceivable that the absolute numbers of circulating DC subsets may fluctuate wildly because of changes in total leukocyte counts in different disease states and errors in operation. In our study, we observed that UAP and STEMI patients had a lower level of circulating mDC2s when compared with controls without coronary plaque. Human mDC2s share most features with murine CD8α + DCs, while mDC1s represent human counterpart of murine CD8α- DCs [22]. In response to viral infections and inflammatory stimuli, mDC1s activates CD4+ T lymphocytes and possess the capacity to promote Treg differetiation [23]. Conversely, mDC2s prime CD8+ T lymphocytes [24]. The mechanisms of decreased circulating mDC2s in CAD are unclear; however, the possible theories have been postulated. In former studies, decreased circulating DCs in CAD were shown to be accompanied with a resident DC increase in the inflamed plaques and infarct myocardium, inferring that circulating DC precursor recruitment to inflamed plaques was a part of the underlying disease process [20, 25]. Additionally, the pathological study showed a high density of resident DCs with frequent T lymphocyte contacts in unstable plaques, suggesting that tissue-resident DCs, at least in part, facilitated local T lymphocyte activation and aggravated atherosclerotic development [26, 27]. Collectively, it is likely that decreased circulating mDC2s in CAD patients are caused by their recruitment into inflamed lesions and infarct myocardium. Subsequently, mature mDC2s in response to inflammatory stimuli within vulnerable plaques promote CD8+ T lymphocyte activation and aggravate atherosclerotic development [27].

According to the severity of symptoms, some published studies grouped CAD patients into patients with stable angina pectoris (SAP) and with acute coronary syndrome (ACS). They reported that mDCs or pDCs were dramatically declined as CAD became more serious [13]. However, these subjects were not classified according to the plaque morphology in their coronary arteries. Although TCFA determined by OCT are found more prevalent in patients with ACS than in those with SAP, there are approximate 20% patients with SAP that have TCFA observed by OCT. [2] In agreement with the previous meta-analysis, the prevalence of TCFA is markedly higher in ACS group than that in SAP group [28]. TCFA harboring in patients with SAP seems to be neglected by clinical presentations but gradually evolve into plaque rupture [29]. Therefore, we applied OCT, which allows for the accurate assessment of plaque characteristics, to objectively identify the different stages of atherosclerosis. It should be noted that plaque rupture detected by OCT accounts for over 40% ACS, while the other ACS are caused by plaque erosion and calcified nodule [30]. As compared with plaque rupture, plaque erosion and calcified nodule are more prevalent in fibrous plaque than those in lipid plaque and TCFA [31]. Therefore, apart from vulnerable plaque characterized as TCFA, culprit plaque erosion and calcification pertain to fibrous plaque also contribute to the incidence of ACS. Additionally, TCFA alone is unable to correctly predict the adverse events in CAD patients. In contrast, TCFA combined with traditional risk factors is referred to be more feasible for evaluate the prognosis of CAD [28]. In patients undergoing coronary angiography, mDC2s were decreased in UAP and STEMI patients when compared with healthy controls, whereas the percentage and absolute number of mDC2s remained similar between UAP and STEMI patients. Moreover, as reported in the OCT subgroup, mDC2s were not significantly altered between patients with and without TCFA. Collectively, these results implied that decreased circulating mDC2s were associated with the presence of coronary plaque but not altered during plaque destabilization and rupture. Though CD11c + DCs were found to reside in vulnerable plaques in mice and humans, it was uncertain whether these resident DCs were derived from circulating common DC precursors or monocytes [25]. Moreover, the specific markers are needed to distinguish the different subsets of resident DCs in coronary plaques. [12]

Current studies unveil that monocyte subsets have different propensities to migrate the vascular wall and differentiate into particular subsets of DCs and macrophages located in plaques. [32, 33] This recognition prompted researchers to determine the association between distinct monocyte subsets and CAD. It is worth noting that some laboratories only referred to two monocyte subpopulations (CD14 + CD16- and CD14 + CD16+ monocytes). The consistent result of these studies was that the count of CD16 positive subsets was higher in patients with CAD and MI as compared with healthy controls.[34, 35] Rogacev et al. [9] found that the count of CD14++CD16+ Mon2 subsets was an independent predictor of adverse cardiovascular events. Tapp et al. [7] reported that circulating Mon2 subsets were prominently elevated in STEMI patients compared with patients with stable CAD and healthy volunteers. Our work extends the results of earlier studies on CAD patients diagnosed by coronary angiography. We showed that elevated CD14++CD16+ Mon2 subsets and reduced CD14 + CD16++ Mon3 subsets were associated with the presence and progression of coronary plaques determined by coronary angiography and OCT. In addition, we found that the proportion of circulating Mon3 subsets were positively correlated with fibrous cap thickness and negatively correlated with arc of lipid core, whereas none of monocyte subsets were significantly correlated with plaque volume. Considering that plaque composition, rather than plaque volume, plays an important role in the plaque disruption and subsequent thrombosis that leads to acute cardiovascular events [1], higher proportion and absolute number of Mon2 may provide important information to predict the presence of TCFA and the progression of atherosclerosis in coronary arteries.

Pathological studies have pushed forward the recognition of human monocyte heterogeneity and biological functions [5, 32]. Mechanistically, the intermediate Mon2 subsets expressing high levels of Tie-2 and CXCR4 possess pro-angiogenic properties [36, 37]. Recent studies uncovered that neovascularization of vasa vasorum within plaques facilitate macrophage infilitration and precipitate plaque vulnerability and rupture [38, 39]. Another important mechanism is that circulating monocytes eventually differentiate into resident DCs/macrophages in atheroma and myocardium, and continue to function during the development of atherosclerosis and myocardial infarction [40]. In this regard, our results revealed that Mon1 and Mon2 were highly related to the serological markers of plaque rupture and myocardial infarction. Murine counterparts of human circulating monocytes could be delineated as Ly-6C^hi^ and Ly-6C^lo^ monocytes. In murine experiments, Ly-6C^hi^ monocytes substantially increased in peripheral blood of Apoe−/− mice fed a high-fat diet and differentiated into pro-inflammatory M1-type macrophages, while Ly-6C^lo^ monocytes differentiated more readily into anti-inflammatory M2-type macrophages [41]. Taken together, these phenomena suggest that both higher Mon2 counts may precipitate plaque destabilization and rupture.

### Study limitations

There were some limitations associated with the present study. First, the study population was relatively small and the study design was cross-sectional in nature. Therefore, we were unable to distinguish whether or not the variation in circulating monocyte subsets is causative of atherosclerotic process. There is a need for prospective studies that investigate the predictive value of circulating DC and monocyte subsets for the development of coronary plaque. Additionally, currently, there was no standardization in the measurement of circulating DC and monocyte subsets.

## Conclusions

Higher proportion and counts of Mon2 subsets are associated with atherosclerosis destabilization and rupture, which may be promising biomarkers for further clinic management.

## Additional files

Additional file 1: Figure S1. Flow chart of the study. OCT = optical coherence tomography, STEMI = ST-segment elevation myocardial infarction, UAP = unstable angina pectoris. (TIFF 903 kb)

Additional file 2: Figure S2. Representative OCT images at culprit lesions. **A.** Fibrotic plaque. The thickness of fibrous cap was 130 μm. An arc delineates the lipid core. Arrows delineate the fibrous cap. **B.** Thin-cap fibroatheroma (TCFA). The thickness of fibrous cap was 60 μm. Arrows delineate the TCFA. **C.** Calcified plaque. (TIFF 8907 kb)

Additional file 3: Figure S3. Representative images showing the identification of circulating DC and monocyte subsets by flow cytometry. Upper panel, identification of DC subsets: mDC1s, mDC2s and pDCs were detected according to the markers. FSC/SSC dot plots were created to exclude debris and platelets. Gated on P1, CD14−/CD19−/SSC dot plots were generated. CD14+ monocytes, CD19+ B lymphocytes and CD14−/CD19−/SSC+ granulocytes were excluded. Gated on P2, CD1c + region was drawn to identify mDC1s and CD141+ region was drawn to define mDC2s. CD303+ region was circled to identify pDCs. Lower panel, identification of monocyte subsets: Mon1, Mon2 and Mon3 were detected according to the markers. Gated on P3 by FSC/SSC, CD45+/CD86 region was drawn to define total monocytes (P4 gate). Mon1, CD14++ CD16- monocytes; Mon2, CD14++CD16+ monocytes; Mon3, CD14+ CD16++ monocytes. FSC = Forward scatter, SSC = Side scatter. (TIFF 10999 kb)

Additional file 4: Table S1. Baseline characteristics of study population. (DOC 41 kb)

Additional file 5: Table S2. Total counts and proportions of DC and monocyte subsets in patients with and without smoking. (DOC 35 kb)

Additional file 6: Table S3. Correlation between glucose and triglyceride and circulating DC and monocyte subsets. (DOC 37 kb)

Additional file 7: Table S4. Correlation between inflammatory markers, myocardial necrotic markers and circulating DC and monocyte subsets. (DOC 45 kb)

Additional file 8: Table S5. Correlation between plaque characteristics and circulating DCs and monocytes. (DOC 40 kb)

Additional file 9: Table S6. Total counts and proportions of DC and monocyte subsets in patients with and without calcified plaque. (DOC 34 kb)

## Abbreviations

CAD: Coronary artery disease
DC: Dendritic cell
Mon: monocyte
OCT: Optical coherence tomography
STEMI: ST-segment elevation myocardial infarction
TCFA: Thin-cap fibroatheroma
UAP: Unstable angina pectoris
