# Supplementary figures and images for: Comparison of circulating dendritic cell and monocyte subsets at different stages of atherosclerosis: insights from optical coherence tomography
Source: BMC Cardiovasc Disord. 2017 Oct 18;17:270. doi: 10.1186/s12872-017-0702-3 (PMC5648428; doi:10.1186/s12872-017-0702-3)

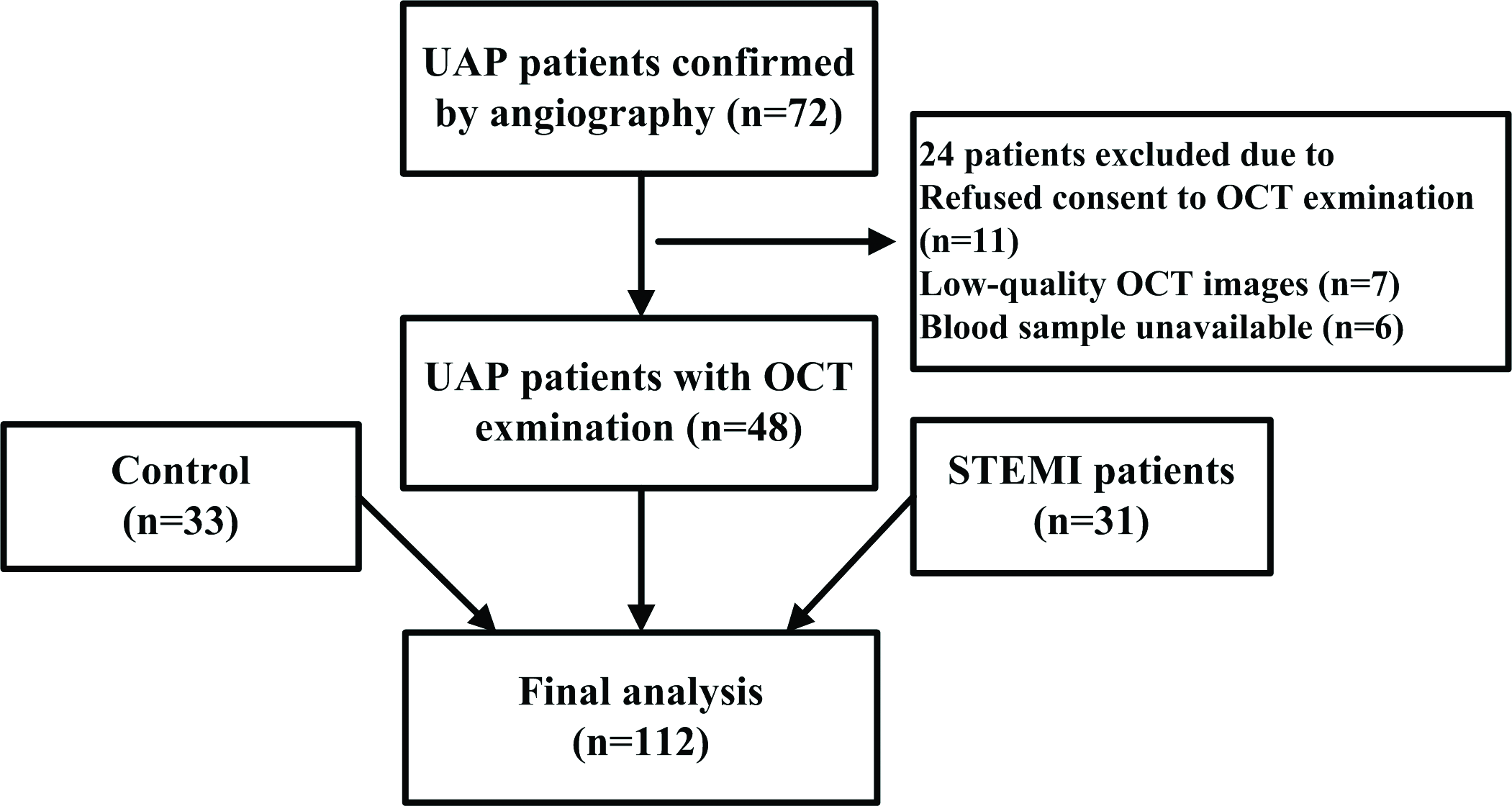

Supplement: Supplementary file 1 — Flow chart of the study. OCT = optical coherence tomography, STEMI = ST-segment elevation myocardial infarction, UAP = unstable angina pectoris. (TIFF 903 kb) [file 12872_2017_702_MOESM1_ESM.tif]

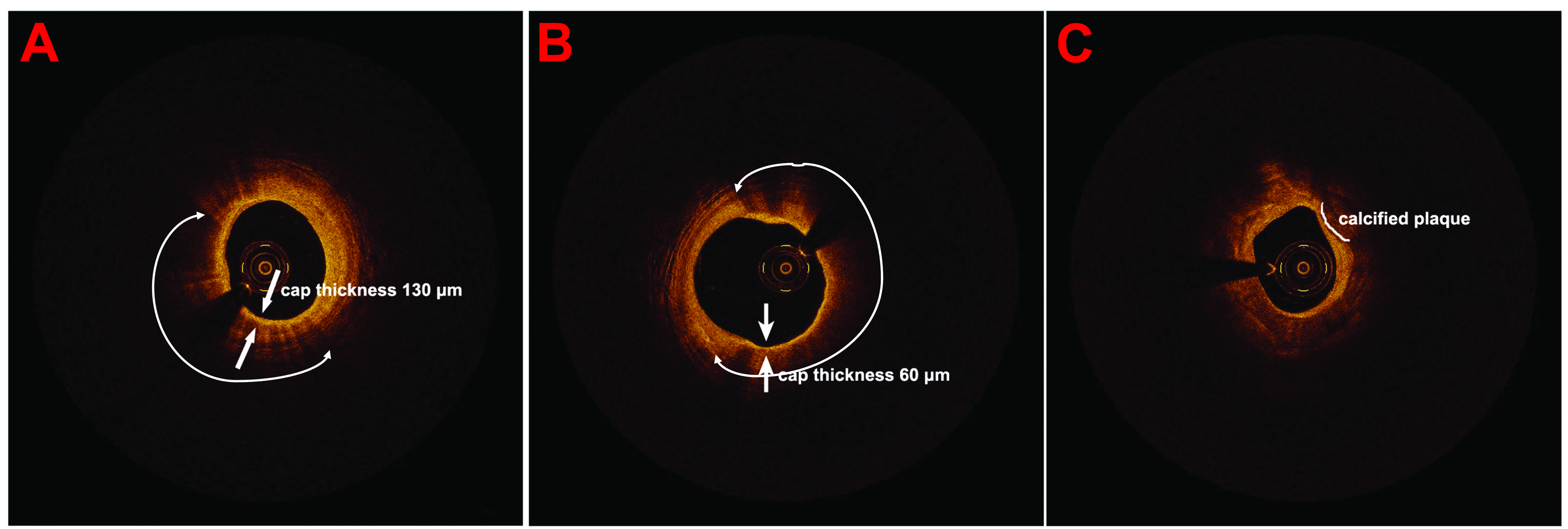

Supplement: Supplementary file 2 — Representative OCT images at culprit lesions. A. Fibrotic plaque. The thickness of fibrous cap was 130 μm. An arc delineates the lipid core. Arrows delineate the fibrous cap. B. Thin-cap fibroatheroma (TCFA). The thickness of fibrous cap was 60 μm. Arrows delineate the TCFA. C. Calcified plaque. (TIFF 8907 kb) [file 12872_2017_702_MOESM2_ESM.tif]

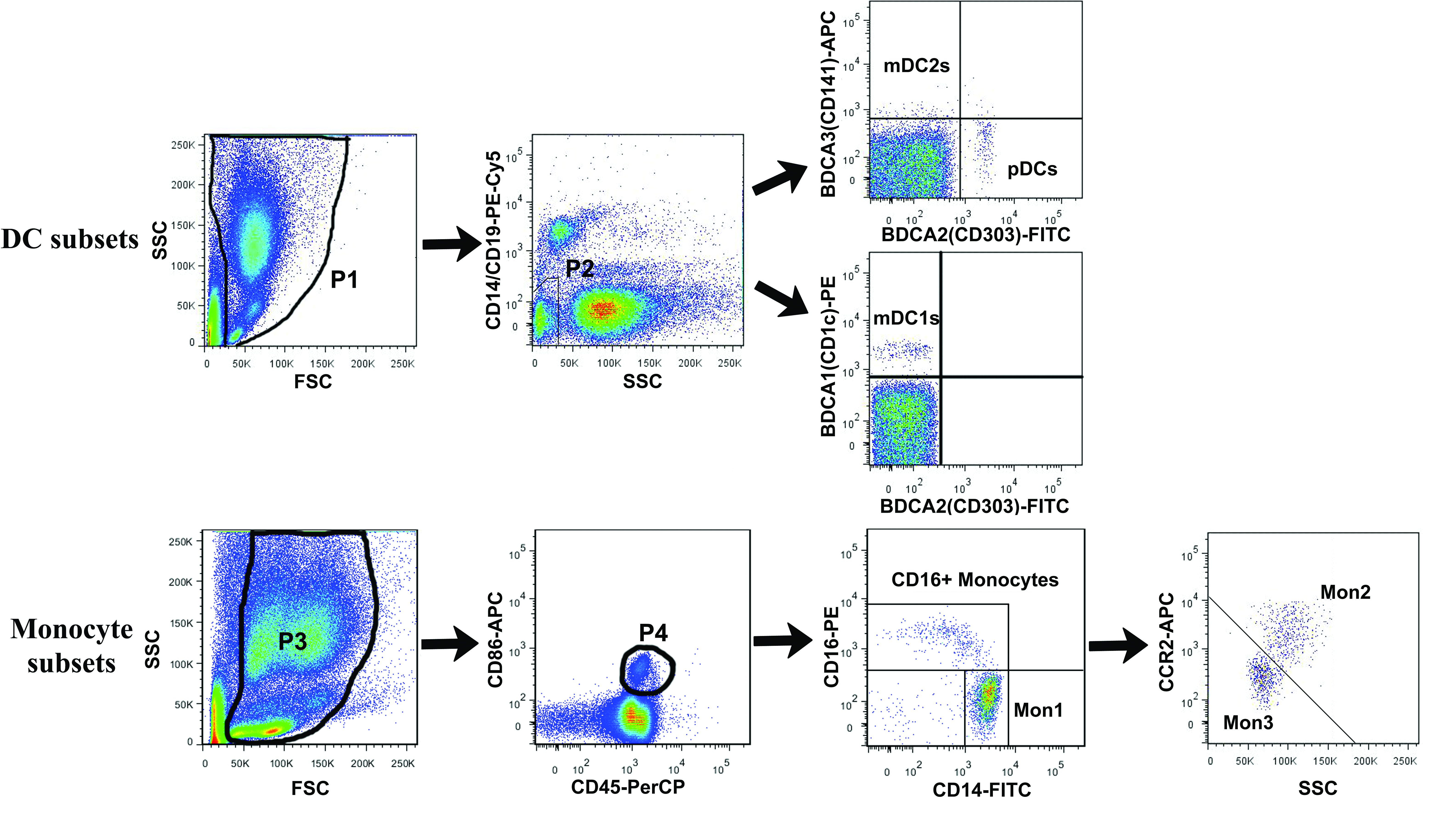

Supplement: Supplementary file 3 — Representative images showing the identification of circulating DC and monocyte subsets by flow cytometry. Upper panel, identification of DC subsets: mDC1s, mDC2s and pDCs were detected according to the markers. FSC/SSC dot plots were created to exclude debris and platelets. Gated on P1, CD14−/CD19−/SSC dot plots were generated. CD14+ monocytes, CD19+ B lymphocytes and CD14−/CD19−/SSC+ granulocytes were excluded. Gated on P2, CD1c + region was drawn to identify mDC1s and CD141+ region was drawn to define mDC2s. CD303+ region was circled to identify pDCs. Lower panel, identification of monocyte subsets: Mon1, Mon2 and Mon3 were detected according to the markers. Gated on P3 by FSC/SSC, CD45+/CD86 region was drawn to define total monocytes (P4 gate). Mon1, CD14++ CD16- monocytes; Mon2, CD14++CD16+ monocytes; Mon3, CD14+ CD16++ monocytes. FSC = Forward scatter, SSC = Side scatter. (TIFF 10999 kb) [file 12872_2017_702_MOESM3_ESM.tif]
