# Supplementary material for: Comparison of circulating dendritic cell and monocyte subsets at different stages of atherosclerosis: insights from optical coherence tomography
Source: BMC Cardiovasc Disord. 2017 Oct 18;17:270. doi: 10.1186/s12872-017-0702-3 (PMC5648428; doi:10.1186/s12872-017-0702-3)
Supplement: Supplementary file 4 — Baseline characteristics of study population. (DOC 41 kb) [file 12872_2017_702_MOESM4_ESM.doc]

**Table S1. Baseline characteristics of study population**

|  | Control (n=33) | UAP  (n=48) | STEMI (n=31) | p value |
| --- | --- | --- | --- | --- |
| Age, yrs | 66.4 ± 10.8 | 61.7 ± 9.0 | 68.3 ± 11.9 | 0.111 |
| Male | 19 (57.6) | 39 (81.3) | 24 (77.4) | 0.050 |
| Smoking | 4 (12.1) | 17 (35.4) | 16 (51.6) | **0.003** |
| Diabetes | 6 (18.2) | 10 (20.8) | 5 (16.1) | 0.869 |
| Fasting glucose, mmol/L | 5.5 ± 2.1 | 5.5 ± 1.8 | 7.4 ± 3.5 | **0.001** |
| Hypertension | 22 (66.7) | 36 (75.0) | 22 (71.0) | 0.715 |
| Triglyceride, mmol/L | 1.7 ± 1.2 | 1.7 ± 1.6 | 3.0 ± 1.4 | **<0.001** |
| Cholesterol, mmol/L | 4.5 ± 1.4 | 4.7 ± 1.7 | 4.5 ± 1.0 | 0.807 |
| LDL-C, mmol/L | 2.7 ± 1.1 | 2.7 ± 0.8 | 2.7 ± 0.9 | 0.632 |
| HDL-C, mmol/L | 1.1 ± 0.3 | 1.1 ± 0.2 | 1.2 ± 0.3 | 0.514 |
| BUN, mmol/L | 5.5 ± 1.6 | 5.6 ± 1.1 | 6.2 ± 2.0 | 0.063 |
| Creatinine, mg/L | 77.9 ± 32.0 | 79.2 ± 12.0 | 88.0 ± 25.2 | 0.180 |
| hs-CRP, mg/L | 1.01 ± 0.42 | 1.39 ± 0.51 | 1.89 ± 0.58 | **0.042** |
| MMP9, ng/ml | 87.2 ± 36.8 | 364.9 ± 100.3 | 433.9 ± 109.3 | **<0.001** |
| Fibrinogen, g/L | 1.31 ± 0.38 | 3.17 ± 0.74 | 3.21 ± 0.82 | **0.018** |
| cTnT, μg/L | 0.02 ± 0.01 | 0.08 ± 0.03 | 0.74 ± 0.33 | **<0.001** |
| CK-MB, ng/ml | 31.5 ± 10.2 | 69.3 ± 15.8 | 302.8 ± 71.1 | **<0.001** |

Values are mean ± SD or n (%).

Abbreviations: BUN, blood urine nitrogen; CK-MB, creatine kinase-MB; cTnT, cardiac troponin T; HDL-C, high-density lipoprotein cholesterol; hs-CRP, high sensitivity C-reactive protein; LDL-C, low-density lipoprotein cholesterol; STEMI, ST-segment elevation myocardial infarction; UAP, unstable angina pectoris.
