# Supplementary material for: Comparison of circulating dendritic cell and monocyte subsets at different stages of atherosclerosis: insights from optical coherence tomography
Source: BMC Cardiovasc Disord. 2017 Oct 18;17:270. doi: 10.1186/s12872-017-0702-3 (PMC5648428; doi:10.1186/s12872-017-0702-3)
Supplement: Supplementary file 5 — Total counts and proportions of DC and monocyte subsets in patients with and without smoking. (DOC 35 kb) [file 12872_2017_702_MOESM5_ESM.doc]

**Table S2. Total counts and proportions of DC and monocyte subsets in patients with and without** smoking

|  | Patients with smoking (n=37) | Patients without smoking (n=75) | p value |
| --- | --- | --- | --- |
| mDC1s, % WBC | 0.22 ± 0.06 | 0.24 ± 0.06 | 0.490 |
| mDC2s, % WBC | 1.01×10^-2^ ± 0.54×10^-2^ | 1.29×10^-2^ ± 0.68×10^-2^ | 0.278 |
| mDCs, % WBC | 0.23 ± 0.06 | 0.26 ± 0.06 | 0.086 |
| pDCs, % WBC | 0.15 ± 0.03 | 0.17 ± 0.05 | 0.758 |
| mDC1s, ×10^4^/ml | 1.69 ± 0.90 | 1.73 ± 0.56 | 0.227 |
| mDC2s, ×10^4^/ml | 0.09 ± 0.05 | 0.08 ± 0.05 | 0.705 |
| mDCs, ×10^4^/ml | 1.76 ± 0.95 | 1.82 ± 0.57 | 0.564 |
| pDCs, ×10^4^/ml | 1.15 ± 0.37 | 1.19 ± 0.38 | 0.620 |
| Mon1, % monocytes | 82.23 ± 5.46 | 81.88 ± 6.08 | 0.762 |
| Mon2, % monocytes | 11.40 ± 3.83 | 9.97 ± 4.26 | 0.087 |
| Mon3, % monocytes | 6.43 ± 4.87 | 8.14 ± 5.36 | 0.103 |
| Mon1, ×10^5^/ml | 3.82 ± 1.05 | 3.66 ± 1.55 | 0.602 |
| Mon2, ×10^4^/ml | 5.19 ± 2.42 | 4.52 ± 3.18 | 0.308 |
| Mon3, ×10^4^/ml | 2.83 ± 2.05 | 3.92 ± 1.49 | 0.118 |

Values are mean ± SD.

Abbreviations: DC, dendritic cell; Lym, lymphocyte; mDC, myeloid dendritic cell; Mon, monocyte; pDC, plasmacytoid dendritic cell; PMNs, polymorphonuclear granulocytes; WBC, white blood cell.
