# Supplementary material for: Comparison of circulating dendritic cell and monocyte subsets at different stages of atherosclerosis: insights from optical coherence tomography
Source: BMC Cardiovasc Disord. 2017 Oct 18;17:270. doi: 10.1186/s12872-017-0702-3 (PMC5648428; doi:10.1186/s12872-017-0702-3)
Supplement: Supplementary file 6 — Correlation between glucose and triglyceride and circulating DC and monocyte subsets. (DOC 37 kb) [file 12872_2017_702_MOESM6_ESM.doc]

**Table S3. Correlation between glucose and triglyceride and circulating DC and monocyte subsets**

|  | Fasting glucose | | Triglyceride | |
| --- | --- | --- | --- | --- |
|  | r | p value | r | p value |
| mDC1s, % WBC | -0.165 | 0.101 | 0.183 | 0.056 |
| mDC2s, % WBC | -0.169 | 0.092 | 0.077 | 0.435 |
| mDCs, % WBC | -0.180 | 0.073 | 0.137 | 0.173 |
| pDCs, % WBC | -0.193 | 0.061 | 0.045 | 0.657 |
| mDC1s, ×10^4^/ml | 0.130 | 0.202 | 0.185 | 0.054 |
| mDC2s, ×10^4^/ml | -0.074 | 0.472 | 0.146 | 0.144 |
| mDCs, ×10^4^/ml | 0.120 | 0.240 | 0.112 | 0.262 |
| pDCs, ×10^4^/ml | 0.124 | 0.219 | 0.015 | 0.881 |
| Mon1, % monocytes | -0.065 | 0.518 | -0.081 | 0.412 |
| Mon2, % monocytes | 0.189 | 0.072 | -0.006 | 0.951 |
| Mon3, % monocytes | 0.093 | 0.376 | 0.115 | 0.246 |
| Mon1, ×10^5^/ml | -0.075 | 0.470 | 0.013 | 0.899 |
| Mon2, ×10^4^/ml | 0.136 | 0.194 | 0.007 | 0.948 |
| Mon3, ×10^4^/ml | 0.083 | 0.391 | 0.099 | 0.339 |

Values are mean ± SD.

Abbreviations as Supplementary Table 2.
