# Supplementary material for: Comparison of circulating dendritic cell and monocyte subsets at different stages of atherosclerosis: insights from optical coherence tomography
Source: BMC Cardiovasc Disord. 2017 Oct 18;17:270. doi: 10.1186/s12872-017-0702-3 (PMC5648428; doi:10.1186/s12872-017-0702-3)
Supplement: Supplementary file 7 — Correlation between inflammatory markers, myocardial necrotic markers and circulating DC and monocyte subsets. (DOC 45 kb) [file 12872_2017_702_MOESM7_ESM.doc]

**Table S4. Correlation between inflammatory markers, myocardial necrotic markers and circulating DC and monocyte subsets**

|  | hs-CRP | | Fibrinogen | | MMP9 | | cTnT | | | CK-MB | |
| --- | --- | --- | --- | --- | --- | --- | --- | --- | --- | --- | --- |
|  | r | p value | r | p value | r | p value | r | p value | | r p value | |
| mDC1s, % WBC | 0.217 | 0.083 | 0.082 | 0.824 | 0.013 | 0.962 | 0.311 | | 0.050 | 0.304 | 0.055 |
| mDC2s, % WBC | 0.194 | 0.183 | -0.118 | 0.608 | 0.155 | 0.716 | **0.491** | | **0.020** | **0.375** | **0.039** |
| mDCs, % WBC | 0.209 | 0.094 | 0.093 | 0.695 | 0.053 | 0.901 | **0.412** | | **0.038** | **0.317** | **0.048** |
| pDCs, % WBC | 0.252 | 0.070 | -0.163 | 0.576 | 0.273 | 0.071 | 0.015 | | 0.964 | 0.016 | 0.961 |
| mDC1s, ×10^4^/ml | 0.248 | 0.079 | 0.113 | 0.627 | 0.032 | 0.912 | 0.308 | | 0.052 | 0.300 | 0.061 |
| mDC2s, ×10^4^/ml | 0.203 | 0.102 | -0.192 | 0.678 | 0.128 | 0.823 | **0.513** | | **0.016** | **0.382** | **0.035** |
| mDCs, ×10^4^/ml | 0.239 | 0.081 | 0.071 | 0.860 | 0.072 | 0.873 | **0.420** | | **0.030** | **0.321** | **0.045** |
| pDCs, ×10^4^/ml | 0.293 | 0.057 | -0.189 | 0.613 | 0.213 | 0.097 | 0.009 | | 0.982 | 0.018 | 0.935 |
| Mon1, % monocytes | **0.483** | **0.023** | 0.313 | 0.059 | **0.569** | **0.003** | **0.515** | | **0.015** | **0.518** | **0.014** |
| Mon2, % monocytes | 0.277 | 0.065 | 0.282 | 0.074 | **0.402** | **0.030** | **0.620** | | **<0.001** | **0.605** | **<0.001** |
| Mon3, % monocytes | -0.238 | 0.083 | -0.183 | 0.595 | -0.301 | 0.054 | -0.048 | | 0.913 | -0.073 | 0.869 |
| Mon1, ×10^5^/ml | **0.537** | **0.010** | 0.283 | 0.074 | **0.618** | **<0.001** | **0.517** | | **0.014** | **0.521** | **0.012** |
| Mon2, ×10^4^/ml | 0.307 | 0.052 | 0.302 | 0.062 | **0.381** | **0.035** | **0.623** | | **<0.001** | **0.691** | **<0.001** |
| Mon3, ×10^4^/ml | -0.268 | 0.068 | -0.165 | 0.572 | -0.218 | 0.069 | -0.047 | | 0.917 | -0.062 | 0.882 |

Values are mean ± SD.

Abbreviations as Supplementary Table 1 and Supplementary Table2.
