# Supplementary material for: Comparison of circulating dendritic cell and monocyte subsets at different stages of atherosclerosis: insights from optical coherence tomography
Source: BMC Cardiovasc Disord. 2017 Oct 18;17:270. doi: 10.1186/s12872-017-0702-3 (PMC5648428; doi:10.1186/s12872-017-0702-3)
Supplement: Supplementary file 8 — Correlation between plaque characteristics and circulating DCs and monocytes. (DOC 40 kb) [file 12872_2017_702_MOESM8_ESM.doc]

**Table S5. Correlation between plaque characteristics and circulating DCs and monocytes**

|  | Fibrous cap thickness | | Arc of lipid core | | Relative stenosis | | MLA | |
| --- | --- | --- | --- | --- | --- | --- | --- | --- |
|  | r | p value | r | p value | r | p value | r | p value |
| mDC1s, % WBC | -0.268 | 0.065 | 0.077 | 0.602 | 0.029 | 0.846 | 0.015 | 0.917 |
| mDC2s, % WBC | 0.185 | 0.208 | -0.230 | 0.115 | <0.001 | 0.999 | -0.244 | 0.094 |
| mDCs, % WBC | -0.248 | 0.089 | 0.057 | 0.703 | 0.028 | 0.848 | 0.036 | 0.809 |
| pDCs, % WBC | 0.166 | 0.260 | -0.111 | 0.453 | 0.191 | 0.193 | 0.138 | 0.350 |
| mDC1s, ×10^4^/ml | -0.123 | 0.411 | -0.241 | 0.102 | 0.030 | 0.841 | -0.097 | 0.510 |
| mDC2s, ×10^4^/ml | 0.100 | 0.504 | -0.059 | 0.692 | 0.006 | 0.971 | 0.168 | 0.259 |
| mDCs, ×10^4^/ml | -0.111 | 0.457 | 0.089 | 0.556 | 0.029 | 0.846 | 0.270 | 0.066 |
| pDCs, ×10^4^/ml | 0.154 | 0.303 | -0.091 | 0.543 | 0.073 | 0.628 | -0.120 | 0.422 |
| Mon1, % monocytes | -0.103 | 0.485 | 0.186 | 0.205 | -0.302 | 0.037 | -0.073 | 0.625 |
| Mon2, % monocytes | -0.223 | 0.127 | 0.073 | 0.622 | 0.207 | 0.158 | 0.148 | 0.315 |
| Mon3, % monocytes | **0.409** | **0.004** | **-0.353** | **0.014** | 0.163 | 0.267 | -0.084 | 0.571 |
| Mon1, ×10^5^/ml | -0.199 | 0.212 | 0.081 | 0.616 | -0.214 | 0.145 | -0.138 | 0.349 |
| Mon2, ×10^4^/ml | **-0.383** | **0.014** | **0.413** | **0.001** | 0.227 | 0.121 | 0.186 | 0.206 |
| Mon3, ×10^4^/ml | 0.255 | 0.107 | 0.265 | 0.094 | 0.201 | 0.170 | -0.022 | 0.893 |

Values are mean ± SD

MLA, minimum lumen area; other abbreviations as Supplementary Table 2.
