# Supplementary material for: Comparison of circulating dendritic cell and monocyte subsets at different stages of atherosclerosis: insights from optical coherence tomography
Source: BMC Cardiovasc Disord. 2017 Oct 18;17:270. doi: 10.1186/s12872-017-0702-3 (PMC5648428; doi:10.1186/s12872-017-0702-3)
Supplement: Supplementary file 9 — Total counts and proportions of DC and monocyte subsets in patients with and without calcified plaque. (DOC 34 kb) [file 12872_2017_702_MOESM9_ESM.doc]

**Table S6**. Total counts and proportions of DC and monocyte subsets in patients with and without calcified plaque

|  | Patients with calcified plaque (n=14) | Patients without calcified plaque (n=34) | p value |
| --- | --- | --- | --- |
| mDC1s, % WBC | 0.22 ± 0.04 | 0.22 ± 0.05 | 0.807 |
| mDC2s, % WBC | 1.10×10^-2^ ± 0.45×10^-2^ | 1.11×10^-2^ ± 0.41×10^-2^ | 0.881 |
| mDCs, % WBC | 0.23 ± 0.04 | 0.23 ± 0.05 | 0.800 |
| pDCs, % WBC | 0.15 ± 0.03 | 0.16 ± 0.03 | 0.605 |
| mDC1s, ×10^4^/ml | 1.58 ± 0.32 | 1.64± 0.59 | 0.696 |
| mDC2s, ×10^4^/ml | 0.08 ± 0.03 | 0.08 ± 0.04 | 0.740 |
| mDCs, ×10^4^/ml | 1.66 ± 0.33 | 1.73 ± 0.61 | 0.688 |
| pDCs, ×10^4^/ml | 1.18 ± 0.36 | 1.14 ± 0.39 | 0.792 |
| Mon1, % monocytes | 81.16 ± 6.27 | 82.86 ± 5.24 | 0.734 |
| Mon2, % monocytes | 10.78 ± 4.97 | 10.54 ± 3.87 | 0.343 |
| Mon3, % monocytes | 7.67 ± 5.42 | 6.69 ± 3.15 | 0.857 |
| Mon1, ×10^5^/ml | 3.34 ± 1.32 | 3.46 ± 1.04 | 0.436 |
| Mon2, ×10^4^/ml | 3.99 ± 1.50 | 4.50 ± 2.74 | 0.755 |
| Mon3, ×10^4^/ml | 2.91 ± 1.94 | 3.26 ±2.135 | 0.519 |

Values are mean ± SD

Abbreviations as Supplementary Table 2.
